# Different Hearts, Different Standards

**DOI:** 10.1016/j.jscai.2025.103999

**Published:** 2025-09-30

**Authors:** Dennis W. Kim

**Affiliations:** Children’s Healthcare of Atlanta, Emory University, Atlanta, Georgia

**Keywords:** congenital heart disease, economic inequity, health care policy

Disparity in care delivery for patients with congenital heart disease (CHD) is well recognized and was presented in a white paper from the combined Society for Cardiovascular Angiography & Interventions (SCAI) Clinical Interests Councils.^1^ In this issue of *JSCAI*, Box et al^2^ present a SCAI position statement illustrating the current complex economic challenges from the proceduralist’s perspective inherent to interventional cardiac care for patients with CHD. These patients receive interventional care that can span the entire age gamut—from prenatal life to late adulthood. This is an area that has been long overlooked for many reasons. The high percentage of underinsurance (predominantly by state Medicaid), inadequate procedural designations and valuations, differential compensation compared with adult interventionalists, and the slow process of cardiac device development are the results.

As the authors note, the financial barriers of Medicaid and underinsurance have an outsized impact on the care of children and adults with CHD. The most recently released April 2025 Centers for Medicare & Medicaid Services (CMS) data estimate 37.3 million children enrolled nationwide in Medicaid and the Children’s Health Insurance Program, which translates to over half of children in the United States covered by Medicaid (41%) or children’s health insurance program (10%).^3^^,^^4^ Individual state statistics for Medicaid coverage for children are available.^5^

It would be fair to say that the national political climate has a significant impact on access to and participation in state Medicaid programs. CMS national coverage guidance for Medicare patients may not be universally applied to all patients with CHD on Medicaid, as these programs are largely managed at the state level. Federal Medicaid law sets broad requirements through CMS, but many decisions and services are left to the determination of individual state Medicaid agencies, creating a varied landscape that has become increasingly difficult to navigate. This has allowed discrepancies to exist such that Medicaid physician fees are substantially less than for Medicare. Adding further complexity, individual states can partition Medicaid coverage via multiple managed care organizations, each with different processes for approval, preauthorization, and claim denials. Not only has basic cardiac care become an increasingly unwieldy process for patients with CHD, access to cutting-edge care technological advancements can be limited. Clinical device trials are imperative for the introduction, adoption, and advancement of new treatment technologies. In Georgia, patient coverage for participation in clinical device trials through the Georgia Medicaid State Health Plan was only approved in 2022.

Proper and accurate valuation of procedures has been a long-standing deficit in congenital interventional cardiology. Unique Current Procedural Terminology (CPT) coding is essential for the appropriate valuation of procedures through the resource-based relative value scale, which then determines the relative value unit of a particular procedure. Inherent in this economic disparity is a historical inadequacy of representation and advocacy for CHD interventions. Without the ability to adequately influence decisions, procedural valuation has remained ambiguous, incorrectly referenced, and has not kept up with technological and procedural advances in the field. Incomprehensibly, the first balloon atrial septostomy for emergency newborn palliation of D-transposition of the great arteries was performed in 1966 yet had a work relative value unit valuation of 0 until 2018! It is hard to believe that such a story could be told for any current interventional cardiac procedure performed in adults. Increasing rejection of relative value scale update committee recommendations by CMS and representation by a singular pediatric (currently noninterventional) member of the relative value scale update committee are existential threats to the practice of congenital interventional cardiology.

Also illustrated are the compensation discrepancies that exist for pediatric interventionalists compared with their adult-focused colleagues. Congenital interventionalists are predominantly affiliated with academic institutions. As the authors illuminate, median compensation of pediatric interventionalists at the full professor rank is one-third less than for adult interventionalists. The difference is even starker for those at lower academic rank. Salary disparity for women in medicine has been repeatedly and consistently described. Accreditation Council for Graduate Medical Education data show a continuing rapid increase in the proportion of women enrolled in pediatric residencies, increasing to 71% in 2021, a relative increase of over 13% from 2007.^6^ Therefore, women will represent the largest demographic characteristic from which the workforce in pediatric interventional cardiology will develop. Without improvements in workforce support and workplace environments, congenital interventional cardiology could face a future critical workforce shortage unlike in other procedural fields.

The impact of congenital cardiac interventions will undoubtedly extend into adult interventional cardiology. An inflection point was reached in the early 2000s, at which time the number of adults living with CHD surpassed the number of children affected. In 2010, adults living with CHD constituted 60% of the total CHD population.^7^ Undoubtedly, with future improvements in cardiac surgery and refinements of new interventional catheterization techniques, interventions will rapidly increase in the adult CHD population with increasing longevity. At many centers, interventional care of these patients continues to be centered at pediatric hospitals, but with increasing development of specialized adult congenital heart centers at adult-focused hospitals, care transition has occurred into an environment where the economic nuances of congenital heart care may not be as readily apparent. Within these health systems, proper congenital procedural coding along with awareness of new CPT codes is typically lacking. As a result, these hospitals generally are challenged with the financial aspects of care for this unique subset of patients.

Much credit for the development of recent congenital-specific coding designations should be given to the SCAI CHD community (including many of the authors of this position paper). New, unique, CHD-specific CPT codes for diagnostic procedures, venography, intracardiac, and extracardiac stenting procedures, and therapies for coarctation of the aorta have been the result of their work. As the home for interventional cardiology, SCAI is uniquely positioned for advocacy for congenital interventional cardiologists. This position paper brings to the surface many of the previously hidden issues faced by congenital interventional cardiologists. SCAI should be loudly applauded for addressing these long-standing challenges.
